# Holistic valorisation of lemon peel into textile materials via fungal chitosan and micro-nano fibrillated cellulose

**DOI:** 10.1038/s41598-025-33086-4

**Published:** 2025-12-20

**Authors:** E. R. Kanishka B. Wijayarathna, Amir Mahboubi, Rebecca Mattsson, Maria-Ximena Ruiz-Caldas, Minna Hakkarainen, Akram Zamani

**Affiliations:** 1 Swedish Centre for Resource Recovery, University of Borås, Borås, SE-501 90 Sweden; 2 Department of Fibre and Polymer Technology, KTH Royal Institute of Technology, Stockholm, SE-100 44 Sweden; 3 Department of Chemistry, Stockholm University, Stockholm, SE-10691 Sweden

**Keywords:** Fungal chitosan, Micro-nanocellulose, Dry gel spinning, Lemon peel, Biopolymers, Monofilaments, Ashby’s plot, Biobased textiles, Biotechnology, Materials science, Microbiology, Nanoscience and technology

## Abstract

Food-waste-derived bio-based materials offer both environmental and economic advantages. We utilised waste lemon peel as substrate to generate value-added materials from chitosan-rich fungal cell wall of *Rhizopus delemar* and purified cellulose from pre-treated solid residues. Nutrient from lemon peel was used for fungal cultivation and the cell wall was isolated from the obtained fungal biomass using mild alkali treatment. The fungal cell wall was used to develop a hydrogel through protonation of amino groups in chitosan by lactic acid addition. This hydrogel served as spinning dope to produce fungal monofilaments using dry gel spinning with a tensile strength of 85 MPa. Simultaneously, cellulose purified from pre-treated solid residues, converted to micro-nanocellulose suspension via mechanical fibrillation and underwent dry gel spinning to produce cellulose monofilaments with a tensile strength of 298 MPa. Cellulose fraction was analysed using XRD, FTIR, TGA, and elemental analyses. The micro- and nanoscale structures of fibrillated cellulose were verified by SEM and AFM. The findings of this study demonstrate a novel holistic valorisation approach for lemon peel waste as a resource for bio-based monofilaments, which could be used as alternatives to commercial fibres in textiles.

## Introduction

 By 2030, the demand for sustainable raw materials in the textile and fashion industry could exceed the supply by as much as 133 million tons^1^. The production of value-added renewable textile materials from food waste is one approach to address this demand. Biopolymers derived via the bioprocessing of food waste are ideally environmentally friendly, compatible with biological systems, and pose minimal harm to the surrounding microenvironment^2^. These processes for deriving biopolymers could increase the environmental and economic sustainability of food waste reuse and renewable material production.

Food waste after processing citrus-family fruits, including lemons and limes, typically corresponds to 50% of the fresh fruit weight^3^, resulting in over 10 million metric tons of waste annually. Failure to manage this waste could lead to decomposition by becoming an inevitable substrate for both bacteria and fungi, and even become a potential producer of greenhouse gases and mycotoxins^4^. Consequently, lemon peel waste has become a compelling subject for research in valorisation applications. It has been tested using different valorisation approaches for the production of, e.g., food additives, pharmaceuticals, packaging, and cosmetics^4^. Lemon peels are rich in cellulose and other nutrients, making them suitable candidates for cellulose-based material production and microbial cultivation. Researchers have reported 6.5–20% sugars and 12.7–23% cellulose^5,6^ in lemon peels. Boluda-Aguilar and López-Gómez^5^investigated pretreatment methods for lemon peels to extract nutrients for fermentation in bioethanol production, whereas Ververis, et al^6^. tested lemon peels as a paper pulp supplement owing to their lignocellulosic content. However, the synergistic application of microbial and cellulose-based processes for lemon peel waste valorisation has not been tested previously. Such a holistic approach would improve the ecological, financial, and diversification aspects of lemon peel valorisation.

Fungal cultivation has been further explored in lemon peel waste through both submerged and solid-state fermentation to produce extracellular enzymes, such as cellulase, xylanase, and pectinase, using different species of *Trichoderma* and *Aspergillus niger*^7,8^. After enzyme extraction, the collected fungal biomass is usually discarded, resulting in waste generation^9^. Yet, if suitably processed, this biomass has a considerable potential for the production of value-added materials. The fungal cell wall contains biopolymers, both fibrous and cementing, which are important for the production of bio-based materials^10^. The filamentous fungus *Rhizopus delemar* belongs to the phylum Mucoromycetes (previously zygomycetes) and has been used in previous studies to produce monofilaments owing to chitosan biosynthesis in the fungal cell wall^11–14^.

In a recent review, Ciriminna, et al^15^. highlighted the sustainability and economic benefits of converting citrus processing waste into micro- and nanocellulose, analysing research trends from 2007 to 2024. Cellulose extraction and nanofiber preparation have been tested using orange^16^ and mandarin peels^17^. Berglund, et al^18^. produced cellulose nanofibers from industrial residues using a cost-effective and efficient process that included the purification of the cellulose fraction by alkali and bleaching treatments, followed by fibrillating the cellulose fraction by mechanical treatment using an ultrafine grinder. Hooshmand, et al^19^. have demonstrated monofilament spinning from mechanically fibrillated and chemically unmodified cellulose using a capillary rheometer. Extracting cellulose from fungal cultivation solid residues and processing it for material development increases the total process efficiency while reducing environmental impact.

Implementing a holistic waste valorisation process, we utilised lemon peel waste to extract nutrients for fungal cultivation and to purify residual cellulose. Through this dual-stream process, lemon peel waste was converted into chitosan-rich fungal cell wall material and purified cellulose, both of which were subsequently used to produce textile monofilaments. This work demonstrates a synergistic approach that integrates food waste valorisation, fungal biotechnology, and bio-based material engineering, offering a distinctive method for producing sustainable textile materials from underutilised food industry by-products.

## Materials and methods

### Microorganisms and other materials

The fungal strain *Rhizopus delemar* CBS 140,950 (Centraalbureau Voor Schimmelcultures, Utrecht, The Netherlands), originally isolated from tempeh, was used^20^. Squeezed lemon peels (LP) from juice production were supplied by Herrljunga Drycker (Herrljunga, Sweden). The as-received lemon peels were crushed into ca. 2 cm × 2 cm pieces using a heavy-duty blender (Waring commercial, USA) and stored at −18 °C until use. Glucose, peptone, agar, sodium hydroxide, lactic acid, sodium chlorite, and Sigmacell cellulose (type 50, 50 μm) were purchased from Sigma-Aldrich.

### Lemon Peel pretreatment

Crushed lemon peel (LP) was pre-treated with hot water (6% w/w) to extract the nutrients, which were used as the substrates for the cultivation of fungi. First, water was heated to 45 °C in a brewing kettle (Digiboil 65 L, KegLand, Australia), and the crushed LP was added. The broth was then kept for 2 h at 45 °C with constant heat and mixed every 15 min using a hand mixer (Robot-Coupe, France). Finally, the liquid fraction was separated using nylon brew bags (Brew bag^®^, USA). The liquid and filtered solid fractions were stored at 4 °C until use for fungal cultivation and cellulose purification, respectively.

### Fungal cultivation

The filtrate from the lemon peel (LP) pre-treatment, after adjusting the pH to 5.5 using 1 M NaOH, was used as the substrate for fungal cultivation. First, the pre-culture was prepared in four 250 mL Erlenmeyer flasks with a 100 mL working volume each. The E-flasks were sterilised in an autoclave (VX95, Systec, Germany) at 121 °C for 20 min. A spore suspension was prepared as the inoculum for the E-flasks. Sterile Milli-Q water (20 mL) was added to a previously prepared *Rhizopus delemar* agar plate (20 g/L glucose, 17 g/L agar, and 4 g/L peptone), and the fungal spores were scraped out. To each E-flask containing 100 mL of substrate, 2 mL of spore suspension was added. The pre-culture was cultivated for 24 h in a water bath shaker at 35 °C and 110 rpm. The substrate for the 26 L reactor was sterilised using the same autoclave, and the reactor was sterilised in situ with steam at 121 °C for 20 min. The sterilised substrate was mixed with the pre-culture under aseptic conditions and pumped into the bioreactor. The cultivation was carried out at 35 °C with 1 VVM aeration for 48 h. Samples were collected from the cultivation broth at 0, 7.5, 24, 30.5, and 48 h to analyse the nutrient consumption and metabolite production by high-pressure liquid chromatography (HPLC) using a hydrogen-based ion exchange column (Aminex HPX-87 H, Bio-Rad, Hercules, CA, USA) operated at 60 °C with 0.6 mL/min of 5 mM H_2_SO_4_ as the mobile phase and a refractive index (RI) detector. At the end of cultivation, the biomass was harvested, washed, and dewatered using brew bags with a pore size of 210 μm (Brew bag^®^, USA). The cleaned biomass was stored in a plastic zip-lock bag at −18 °C until use.

### Alkali treatment of fungal biomass and spinning dope Preparation

The biomass was subjected to alkali treatment, as described in detail in our previous study^13^, and is briefly explained here. The biomass was suspended in water at a concentration of 2%. The suspension was then passed through an ultrafine grinder (Mazuko, Japan) at 2700 rpm with a + 50 μm open gap between the grinder stones. NaOH was then added to a final volume of 50 mL, 0.2 M NaOH per gram of dry fungal biomass. This mixture was heat-treated in an autoclave at 121 °C for 20 min. The alkali-insoluble fraction, which mainly consisted of fungal cell wall material, was filtered out and washed to achieve a neutral pH.

The collected fungal cell wall fraction was mixed with dilute lactic acid (0.5 M), and the pH was adjusted to 3 to convert the slurry into a dilute hydrogel. The hydrogel was then homogenised by passing it three times through the same ultrafine grinder at 2700 rpm and a 50 μm gap between the grinder stones. Finally, the hydrogel concentration was adjusted to 4% using a vacuum-filtration unit. This concentrated hydrogel was used as the spinning dope to produce monofilaments using the dry gel spinning method.

### Composition analysis of cell wall material

The composition of the cell wall material collected after alkali treatment was analysed using the method described by Mohammadi, et al^21^.. First the cell wall fraction was freeze dried using a freeze dryer (Labconco FreeZone 84 C, Labconco Corporation, USA) and powdered using a ball mill (Retsch, Germany). The samples were subjected to two-stage acid hydrolysis with 72% V/V sulfuric acid at room temperature for 90 min and with diluted sulfuric acid and heating at 121 °C for 1 h using an autoclave (VX95, Systec, Germany). A liquid sample was taken after the autoclave to measure the concentration of acetic acid released from the cell wall material due to deacetylation reaction. An aliquot of 0.5 ml from the rest of the sample was further treated with 0.5 ml of 1 M sodium nitrite and kept overnight to convert glucosamine residues to 2,5-anhydromannose. On the following day, this sample was treated with 0.5 ml of 12% sodium sulfamate. At the end this sample and the sample collected after the autoclave step were analysed using a hydrogen-based ion exchange column (Aminex HPX-87 H, Bio-Rad, Hercules, CA, USA) operated at 60 °C with 0.6 mL/min of 5 mM H_2_SO_4_ as the mobile phase. The 2,5- anhydromannose measured (with refractive index detector) was corresponding to both glucosamine and N-acetyl glucosamine residues in the chitin and chitosan available in the cell wall and the acetic acid measured (with ultraviolet detector) is corresponding to only N-acetyl glucosamine content. The percentages of chitin and chitosan were determined using the subtraction method, assuming that chitosan consists solely of glucosamine units and chitin consists solely of N-acetylglucosamine units.

### Cellulose purification from the solid residues

After the pre-treatment, lemon peel solid residuals were further processed to purify the cellulose fraction, according to the method presented by Berglund, et al^18^.. The process was as follows: First, the solid residual fraction was washed with Milli-Q water (2%, 10 L suspension dry weight basis) at 85 °C for 2 h. The water was then purged, and the solids were subjected to alkali treatment with 2%, 10 L NaOH at 80 °C for 2 h. After thorough washing and filtration to neutral pH, bleaching was conducted with 1.7% NaClO_2_ in acetate buffer (pH 4.5) at 80 °C for 2 h. For this, the remaining solids after alkali treatment were mixed with 1 L each of 1.7% NaClO_2_, acetate buffer (pH 4.5), and milli-Q water. The solid remaining after bleaching was washed thoroughly until a neutral pH was attained. The remaining pulp was white.

The total valorisation efficiency of lemon peel is calculated using below equation with the assumption of both cell wall and purified cellulose fractions consists of only biopolymers,

1 $$Total\;valorisation\;efficiency = \frac{{Cell\;wall\;material\;\left( g \right) + Purified\;cellulose\;fraction\;\left( g \right)}}{{Starting\;weight\;of\;lemon\;peel\;\left( g \right)}}$$

 

### Characterisation of purified cellulose

The purified fibrillated cellulose fraction was characterised to analyse its purity. The as-received lemon peel and Sigmacell cellulose were also analysed as control samples from both ends. The samples were freeze-dried using a freeze dryer (Labconco FreeZone 84 C, Labconco Corporation, USA) and powdered using a ball mill (Retsch, Germany). To analyse the infrared (IR) spectra, a Fourier-transform infrared spectrometer (FTIR) (Nicolet™ iS50, Thermo Fisher Instruments) was used between the wavelengths 400 to 4000 cm^− 1^. The thermal decomposition was analysed using a thermogravimetric analyser (TGA) (TA instruments, USA) for duplicate samples of ca. 10 mg from 35 °C to 650 °C with 5 °C/min ramping in nitrogen atmosphere. Elemental analyses of the duplicate samples were performed using a Thermo Fisher Scientific CHNS elemental analyser (Thermo Fisher Scientific Inc., USA). The oxygen content was calculated using the subtraction method^22^. The ash contents of the purified fibrillated cellulose and the as-received lemon peel were measured using the TAPPI T 211 om-02 ^23^ method. X-ray Powder Diffraction (XRD) measurements were conducted using a Panalytical X’pert Pro diffractometer (Cu Kα1,2, λ1 = 1.540598 Å, λ2 = 1.544426 Å) in Bragg − Brentano geometry, operated at 45 kV and 40 mA in reflection mode. For Sigmacell cellulose, lemon peel, and its extracted cellulose samples, pellets of 8 mm diameter were prepared by pressing approximately 60 mg of the solid sample at 50 MPa for 15 s. For micro-nanocellulose (the fibrillated cellulose as explained in the section “Mechanical fibrillation of the purified cellulose pulp”), approximately 15 g of a 0.86 wt% cellulose suspension was centrifuged at 35,000 g for 10 min to concentrate it. After removing the excess water, a small amount of the resulting paste was placed on a zero-background silicon wafer using a spatula and left to dry in a fume hood. Diffraction patterns were collected over a 2θ range of 5–50° with a step size of 0.033° and a scan speed of ~ 0.02°/s. A beam knife slit was positioned above the sample, and the sample was rotated with a revolution time of 2 s during acquisition. Background removal was carried out using Fityk peak-fitting software^24^ using a line connecting the two points at 2θ = 8° and 45°.

The crystallinity index (CI) of the sample was calculated using the peak height method developed by Segal, et al^25^. using the following equation:

2 $$CI\left( \% \right) = \frac{{{I_{002}} - {I_{AM}}}}{{{I_{002}}}}$$

Where_002_is the intensity of the peak at 2θ = 21.8° corresponding to the plane 200, andthe minimum intensity between the 200 and the 110 peaks, corresponding to the amorphous phase.

### Mechanical fibrillation of the purified cellulose pulp

The cellulose pulp was diluted to 1% with Milli-Q water, and the suspension was mechanically treated with the same ultrafine grinder (Masuko, Japan) at 2700 rpm to fibrillate the cellulose at the nanoscale. The gap between the grinding stones was gradually decreased from + 50 μm (open gap) to −100 μm (contact mode). Grinding was performed continuously using a custom-made closed-loop pumping system. Samples were collected at several time points, and the viscosity of the diluted pulp was measured using a viscometer (A&D Vibro Viscometer SV-10, A&D Company, Japan). Grinding was stopped when the viscosity reached a plateau, indicating the fibrillation of cellulose^18^.

### Cellulose spinning dope Preparation

The cellulose suspension collected at the end of the fibrillation process was selected to prepare the cellulose spinning dope. The pulp concentration was increased from 1% to 4% using a centrifuge (Heraeus Megafuge 8, Thermo Scientific, USA). The centrifugation was done 2 times at 10,000 g for 30 min. This concentrated cellulose pulp was used as the spinning dope for monofilament production without subjecting it to any chemical modification.

### Dry gel spinning

The dry gel spinning technique described by Lindh, et al^26^. was used to produce continuous monofilaments from the prepared spinning dopes of fungal cell wall hydrogel and fibrillated cellulose suspension. The gel was extruded using a syringe with a needle (0.8 mm Ø) and a syringe pump (New Era Pumps, USA). The extruded monofilaments were collected directly onto a rotating roller covered with a parafilm (Amcor, Switzerland) layer. The monofilaments were dried overnight at room temperature, collected, and stored in plastic bags until further testing.

### Microscopy

To analyse the degree of fibrillation of the micro-nanocellulose and the morphology of the produced monofilaments, the samples were observed using a scanning electron microscope (SEM). Imaging was conducted using an ultra-high-resolution field-emission SEM (S-4800, Hitachi, Tokyo, Japan) operated at an accelerating voltage of 1.0–3.0 kV. For SEM analysis, freeze dried samples were mounted on stubs using carbon tape and coated with a 3 nm layer of Pt/Pd. Furthermore, the monofilaments and their cross-sections were observed using an optical microscope (Nikon Eclipse LV150NL) and NIS-Elements BR 5,41,01 64-bit software. The cross-section of the cellulose monofilament was coloured with a highlighter pen to obtain a clearer image.

The morphology of the micro-nanofibrilated cellulose was further evaluated using a Multimode 8 atomic force microscopy (AFM) instrument (Bruker, USA). Imaging was performed in peak force tapping mode with the aid of the ScanAsyst™ auto-optimization function. For sample preparation, a 0.002 wt% aqueous suspension as first sonicated for 10 min and then some drops were deposited onto a freshly cleaved mica substrate and allowed to dry in a fume hood. The obtained images were analysed with Gwyddion 2.60 software^27^.

### Mechanical properties

The mechanical properties of the monofilaments produced from both cellulose and fungal cell wall hydrogels were tested using tensile testing equipment (H10KT, Tinius Olsen, USA). The moving gauges were initially set to a length of 20 mm. The diameter of the monofilaments was measured using optical microscopy (Nikon, Japan) and NIS Elements V 5.11.01 software (Nikon, Japan). The crosshead speed was set to 1 mm/min, and the preload was set to 0 N. The tensile strength and elongation % at break were obtained using the Horizon software (Tinius Olsen, USA). Before the test, all samples were preconditioned at 23 ± 2 °C and 50 ± 4% relative humidity overnight (ISO 139, 2005).

### Statistical data analysis

Statistical data analyses were performed using Minitab 21 (Minitab^®^ 21.1.1). The significance between the two compared values was tested using a two-sample t-test with a 95% confidence interval. Unless specified otherwise, all analyses were conducted with at least three replicates.

## Results and discussion

**Fig. 1:**
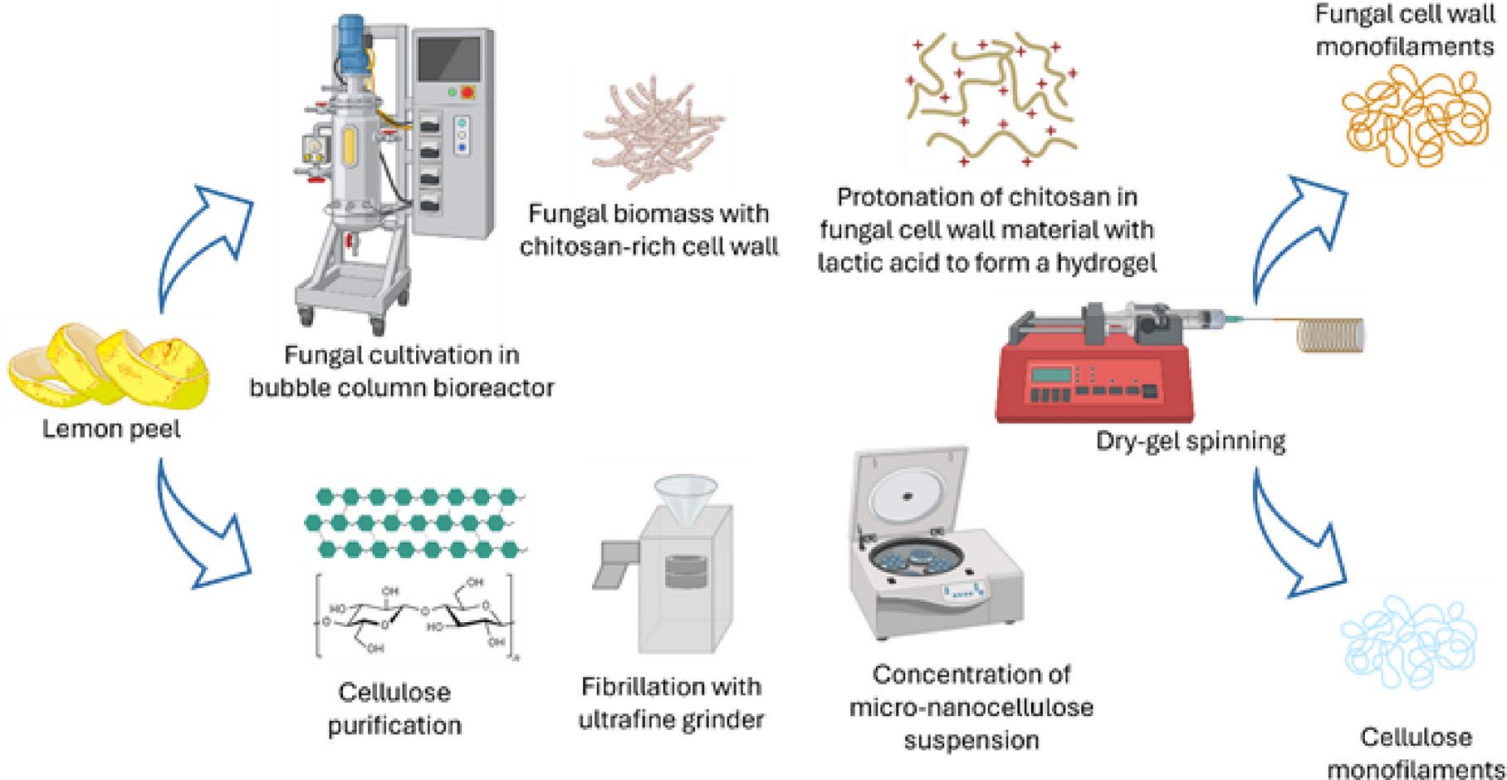
The schematic on the whole process of lemon peel valorisation towards micro-nanocellulose and fungal monofilaments.

Producing biomaterials by valorising organic waste provides a win-win solution for both renewable material production and waste recycling^28^. In this study, lemon peels (LP) from juice production were used as organic waste for valorisation into bio-based textiles (Fig. 1). Fungal cultivation was performed on the extracted nutrient fraction, and the solid residues were further processed to purify cellulose. Fungal cell wall material was isolated from the harvested fungal biomass. Mechanical treatments were performed separately on both the cellulose and cell wall fractions using ultrafine grinding. Monofilaments were produced from both cellulose and fungal cell wall-based hydrogels using a dry gel spinning method. The mechanical properties of the produced monofilaments were analysed, and the results demonstrated the potential of valorising lemon peel waste for the production of bio-based textile materials.

### Lemon Peel pretreatment

**Fig. 2:**
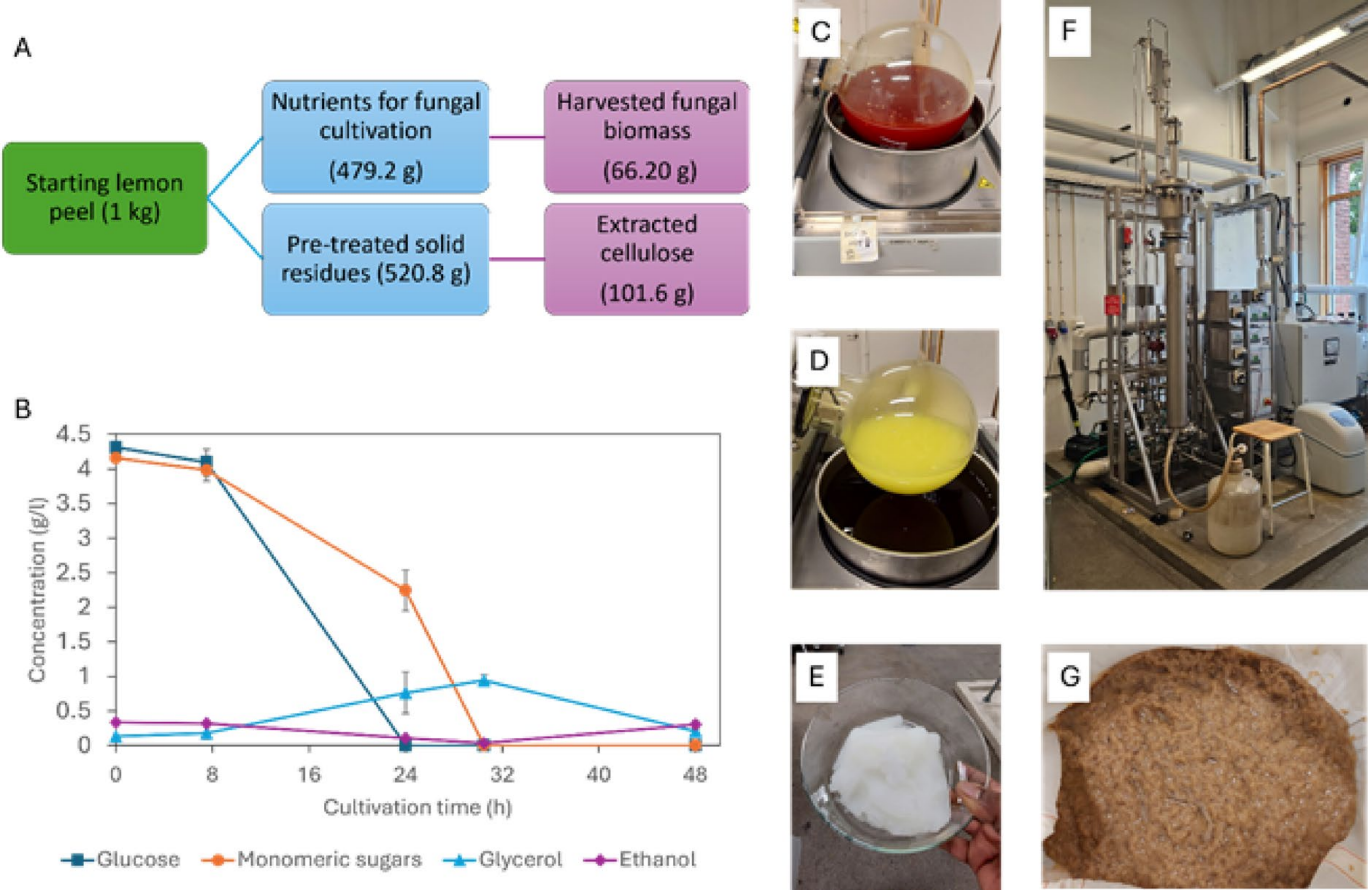
**A** – Summary of the final yields of fungal biomass and cellulose fractions calculated starting from 1 kg of lemon peel waste. **B** – Changes in glucose, monomeric sugars other than glucose, glycerol, and ethanol content of the cultivation media over 48 h. **C** – Lemon peel solid residue alkali treatment. **D** – Bleaching of lemon peel solid residues. **E** – Purified cellulose. **F** – 26 L bubble column bioreactor. **G** – Harvested fungal biomass.

Lemon peel (LP) was pre-treated with water and heated to extract consumable nutrients for the cultivation of fungi. LP pre-treatment was performed in a 25.4 L batch with 6% lemon peels. After 2 h of treatment, the filtered liquid fraction contained 3.6% total solids, and the filtered volume was 20.4 L resulting in an extraction yield of 47% (Fig. 2A).

### Fungal cultivation on the liquid fraction

After 48 h of cultivation in the 26 L bubble column bioreactor (Fig. 2F), the biomass yield was 0.14 g biomass/g substrate (Fig. 2A). The pH was 4.6 at the harvest. The harvested fungal biomass is shown in Fig. 2G. In our previous study, *R. delemar* was cultivated in a 4% bread waste powder suspension with a final biomass concentration of 0.15 g biomass/g substrate, which was marginally higher than the yield obtained in this study^13^. However, this could be due to unconsumed solid bread particles entangled within the biomass^13^. The initial substrate, the liquid fraction collected from the lemon peel pre-treatment, contained 4.31 g/L glucose and 4.16 g/L of other monomeric sugars (Fig. 2B). At 7 h of cultivation, both glucose and other monomeric sugar concentrations decreased, confirming active fungal growth. After 24 h, all the glucose and almost half of the monomeric sugars were consumed. This is a common phenomenon in which organisms prefer glucose over other sugars because of the ease of consumption^29^. The glycerol concentration increased until 30.5 h, indicating the metabolic activity of *Rhizopus delemar*. After 30.5 h, ethanol production occurred as a stress response due to depleted oxygen mass transfer owing to the thicker cultivation broth^30^.

### Fungal cell wall material Preparation from fungal biomass

The fungal strain *Rhizopus delemar* has the ability to biosynthesise chitosan in the cell wall^12,14,31,32^. The cleaned biomass collected during the harvesting step was alkali-treated with NaOH and heated to isolate the cell wall material. The fungal cell wall material was collected after pH neutralisation and appeared as a paste. After alkali treatment, cleaning, and neutralisation, the material yield was 0.28 g/g of biomass, containing 18% N-acetyl glucosamine and 35% glucosamine polymers representing chitin and chitosan. In their research, Svensson, et al^14^. reported an alkali-insoluble material representing the fungal cell wall fraction with a yield of 0.23 g/g biomass, containing 23% chitin and 36% chitosan when the same fungus, *Rhizopus delemar*, was cultivated on bread waste.

### Cellulose purification from the solid residues

Cellulose purification was done using lemon peel-pre-treated solid residues (Fig. 2C, D, and E). The cellulose yield is shown in Fig. 2A. From each gram of pre-treated solid residue, 0.195 g of purified cellulose was extracted, resulting in a yield of 19.5%. The yield was higher than the 12.7% and 15% reported in previous research on lemon and orange peels, respectively^6,16^. However, enzymes were used to remove proteins and pectin in both studies. Berglund, et al^18^. reported higher cellulose yields of 32% and 22% in carrot pomace and brewers’ spent grain, respectively, using the same extraction method. The total valorisation efficiency of the process, expressed as the total amount of recovered biopolymer material per gram of lemon peel waste, is 0.12 g biopolymers/g lemon peel in the present work (Eq. 1).

**Fig. 3:**
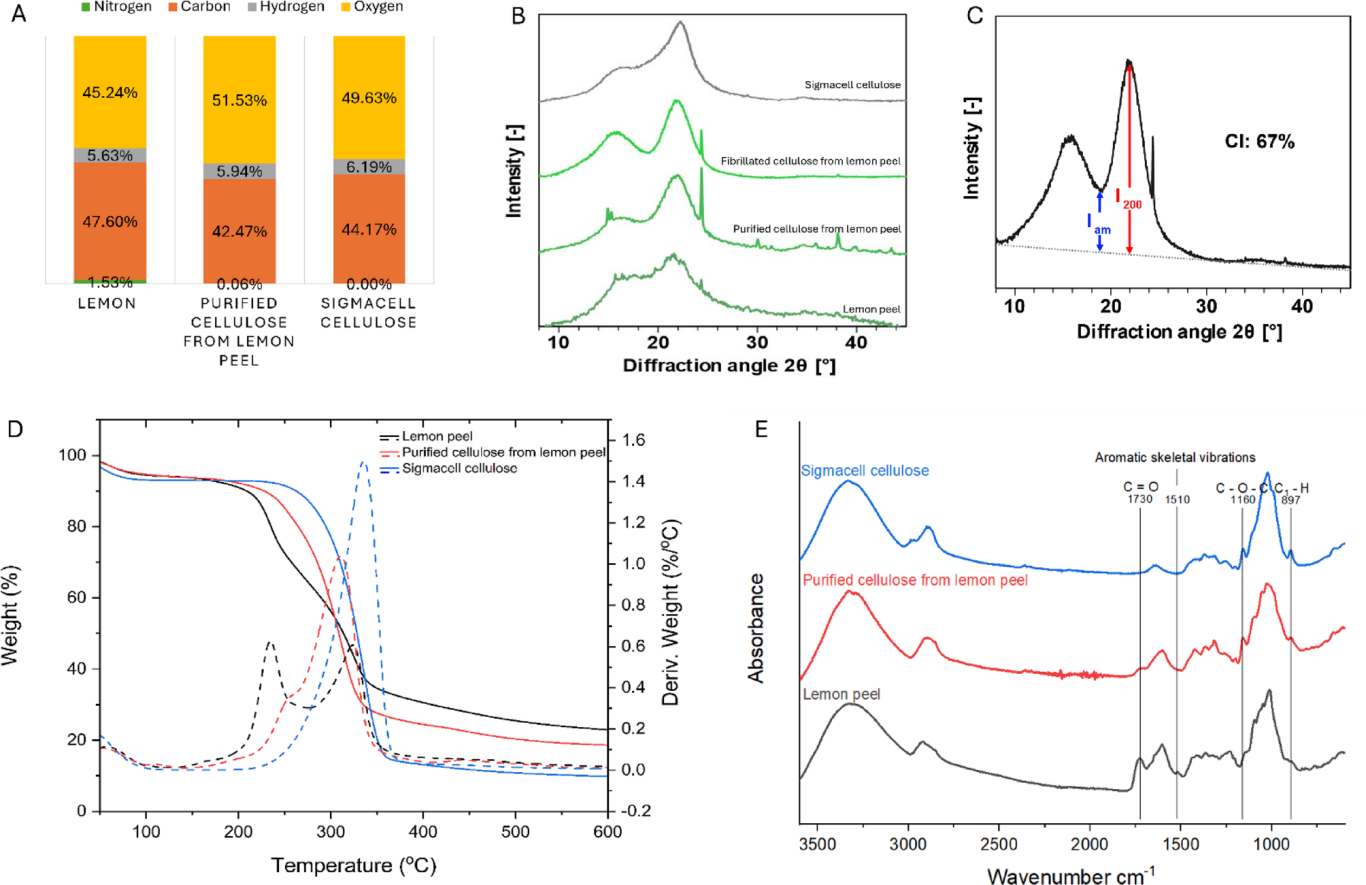
**A** – Elements Nitrogen, Carbon, Hydrogen, and Oxygen (calculated by subtraction) from the elemental analysis. **B** – X-ray diffraction pattern of lemon peel, purified cellulose from lemon peel, fibrillated cellulose from lemon peel and Sigmacell cellulose. **C** – XRD diffraction pattern of the extracted micro-nanocellulose showing the intensities used to calculate the CI by the Segal method. The grey dashed line corresponds to the background used for baseline correction. **D** – Thermogravimetric analysis (TGA) curves. The solid lines show the weight% change, and the dashed lines show the derivative of the weight% per °C. **E** – FTIR spectra of Sigmacell cellulose, purified cellulose from lemon peel, and lemon peel.

The ash contents of the purified cellulose and as-received lemon peel, according to the TAPPI T 211 method, were 4.1 ± 0.1% and 3.4 ± 0.1%, respectively. In their work, Ververis, et al^6^. reported the cellulose and ash contents of lemon peels after pectin extraction as 12.7% and 1.9% respectively. If these two are fractionated to calculate the ash content in cellulose, it results in a 15% ash content in the cellulose fraction. However, it would be incorrect to assume that all the ash from lemon peels will remain after the bleaching process. Sarkar, et al^33^. explained that the interactions between inorganic ions, such as Na^+^, K^+^, and Ca^+^, which contribute to ash content, and hemicelluloses are stronger than those with cellulose and lignin. These inorganic compounds that interacted with hemicellulose in lemon peel could have been washed away during the purification process of lemon peel. This further confirms the lower ash content obtained compared to the calculated ash content of the native lemon peel reported in the literature.

Figure 3: A – Elements Nitrogen, Carbon, Hydrogen, and Oxygen (calculated by subtraction) from the elemental analysis. B – X-ray diffraction pattern of lemon peel, purified cellulose from lemon peel, fibrillated cellulose from lemon peel and Sigmacell cellulose. C – XRD diffraction pattern of the extracted micro-nanocellulose showing the intensities used to calculate the CI by the Segal method. The grey dashed line corresponds to the background used for baseline correction. D – Thermogravimetric analysis (TGA) curves. The solid lines show the weight% change, and the dashed lines show the derivative of the weight% per °C. E – FTIR spectra of Sigmacell cellulose, purified cellulose from lemon peel, and lemon peel.

Elemental analysis of the purified cellulose from lemon peels further elucidated the composition of the sample (Fig. 3A). The results of the elemental analysis show the composition of the organic fraction only and were further rectified by ash% correction. The reference Sigmacell cellulose contained only carbon, hydrogen, and oxygen. The native lemon peel contained 1.5% nitrogen and 47.6% carbon. This nitrogen could be due to the trace amounts of proteins available in lemon peel and compounds such as alkaloids, which give the bitter taste of the lemon peel^34,35^. After purification, the nitrogen content was significantly reduced (*p* = 0.031) to 0.06%. Other elemental compositions, such as carbon and hydrogen, were slightly lower but closely related to those of Sigmacell cellulose. These results show that the purification step of cellulose was successful; however, the results also confirm that the recovered pulp was not 100% cellulose.

The thermal degradation behaviour of the samples was analysed using a thermogravimetric analyser (TGA) to observe the distinct decomposition events related to cellulose, hemicellulose, and lignin, thus further confirming the effectiveness of the cellulose purification step^36^ (Fig. 3D). Sigmacell cellulose exhibited the typical thermal degradation behaviour of cellulose, with a sharp degradation peak at 350 °C and a residual weight of less than 10%^37,38^. The purified cellulose from lemon peel showed the highest thermal degradation at 310 °C and also contained a minor degradation peak at 250 °C which can be attributed to the residual hemicelluloses from the purification process^39^. The as-received lemon peel TGA graph contains two well-visible degradation peaks related to the hemicellulose and cellulose fractions. These observations are consistent with the thermal degradation data of citrus peels reported by Chandrasekar, et al^40^..

The purity of the cellulose was further analysed using FTIR, with Sigmacell cellulose as the reference (Fig. 3E). The obtained FTIR spectra show peaks in both the Sigmacell cellulose and purified cellulose from lemon samples at 897 cm^− 1^ and 1160 cm^− 1^, which are related to the C_1_ – H bending and C – O – C stretching in the β−1,4 glycosidic bond in cellulose^41–43^. These two peaks are not visible in the lemon peel spectrum due to the complex polymeric arrangement in the native form. The peaks at 1510 cm^− 1^ and 1730 cm^− 1^, which are related to the aromatic skeletal vibrations of lignin^41,42,44^ and C = O stretching vibrations of carboxyl and acetyl groups in hemicellulose^41,42^, respectively, are visible only in the lemon peel spectrum. These observations confirm the availability of lignin and hemicellulose in lemon peels and the effectiveness of cellulose purification. Ververis, et al^6^. have reported hemicellulose and lignin amounts in lemon peel after pectin removal as 5.3% and 1.7% respectively. Since these components were present in very low amounts and were not relevant to the scope of this study, they were not analysed.

The crystallinity of the purified cellulose from lemon peel and micro-nanocellulose (fibrillated cellulose explained in section “Mechanical treatment of cellulose to make micro-nano cellulose”) was analysed with X-ray diffraction (XRD) using lemon peel and Sigmacell cellulose as references. The X-ray diffractograms (Fig. 3B and C) of the lemon peel shows a broad pattern with a peak centred around 2θ = 22°, while the purified cellulose from lemon peel and micro-nanocellulose exhibit the typical reflections of cellulose I, with a broad peak at 2θ ≈ 21.8°, corresponding to the (200) plane, and another at 2θ ≈ 15.9°, arising from the overlap of the (1ī0) and (110) planes. The crystallinity indices (CI), determined by the Segal method after background subtraction, were 62% for the purified cellulose from lemon peel and 67% for the fibrillated micro-nanocellulose. The CI of the Sigmacell cellulose used in this study was 54%.

In all lemon-related samples (Fig. 3B and C), a sharp diffraction signal is observed at 2θ = 24.4°, and in the purified cellulose from lemon peel, additional peaks are visible at 14.9°, 30.1°, and 38.2°. These reflections do not originate from cellulose and are attributed to trace amounts of insoluble inorganic crystals in the lemon peel, most likely calcium oxalate monohydrate^45,46^ that accumulated on the cellulose after extraction. Nevertheless, the integrated area of these peaks is negligible compared with the cellulose reflections, indicating that these inorganic crystals are present only at trace levels.

### Mechanical treatment of cellulose to make micro-nano cellulose

**Fig. 4:**
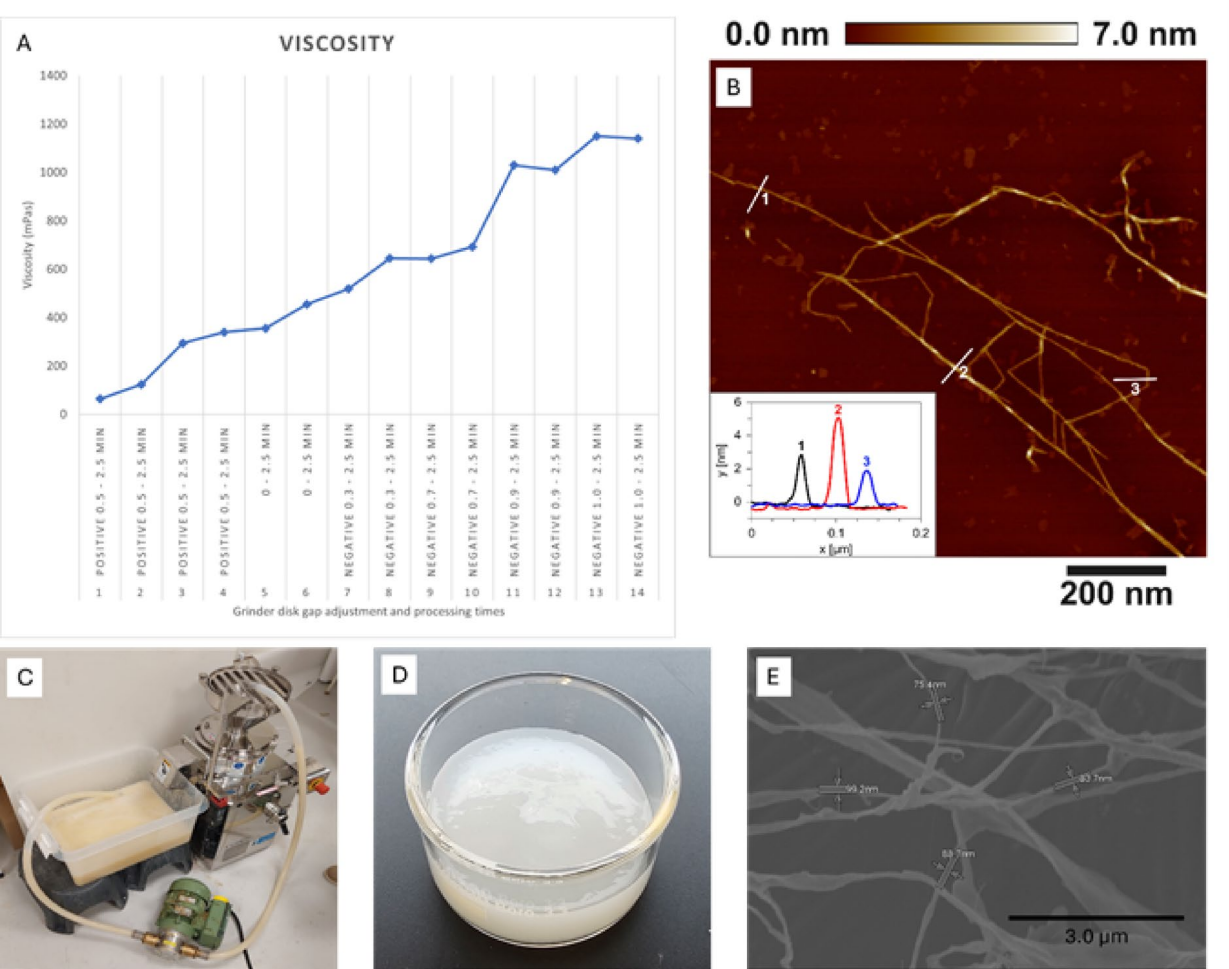
**A** – Viscosity measurements (Positive 0.5 = 5 μm gap between the grinding disks, 0 = the grinding disks are touching each other, and negative gap means the grinding disks are pressed towards each other), **B** – AFM image of nanofibrillated cellulose, **C** – The custom-made closed-loop pumping system incorporated with ultrafine grinder. **D** – fibrillated cellulose suspension. **E** – AFM image of the fibrillated cellulose fraction.

The purified cellulose fraction was fibrillated into micro-nanocellulose via mechanical treatment using an ultrafine grinder (Fig. 4C). The viscosity of the suspension after 2.5 min of grinding with a 50 μm open gap between the disks was 64 mPas. The viscosity was measured at regular intervals of 2.5 min, and the gap between the grinding stones was reduced stepwise (Fig. 4A). The viscosity gradually increased to a plateau at approximately 1145 mPas after 35 min of grinding. The final gap between the stones was negative 100 μm, indicating that the stones were pressed against each other. This gradual increase in viscosity indicates the disintegration of cellulose fibrils^18^. This increase in viscosity can be attributed to the formation of a stronger nanocellulose network^18,47^. Figure 4D shows the fibrillated cellulose suspension. The scanning electron microscope (SEM) image of a freeze-dried suspension (Fig. 4E) contains both micro-and nanoscale structures. Moreover, atomic force microscopy (AFM) image (Fig. 4B) shows high-aspect-ratio fibrils with heights between 2 and 7 nm. Both elementary fibrils and bundles of 2–3 elementary fibrils are observed, indicating successful fibrillation. Both imaging techniques further confirm the micro-nano fibrillation of the purified cellulose by mechanical treatment.

### Spinning dope preparation and dry gel spinning

To prepare the spinning dope for the fungal cell wall fraction, a dilute lactic acid solution (0.2 M) was added to the fungal cell wall fraction collected from the alkali treatment until a pH of 3 was achieved. When the pH reached 3, the material began to behave as a gel. This gelling behaviour can be explained by the protonation of the amino groups in chitosan available in the fungal cell wall. The protonation of amino groups in chitosan that occurs at acidic pH (< 6.0) enables the diffusion of water molecules into the polymer matrix, thus improving the solubility of the macromolecule^48,49^.

**Fig. 5:**
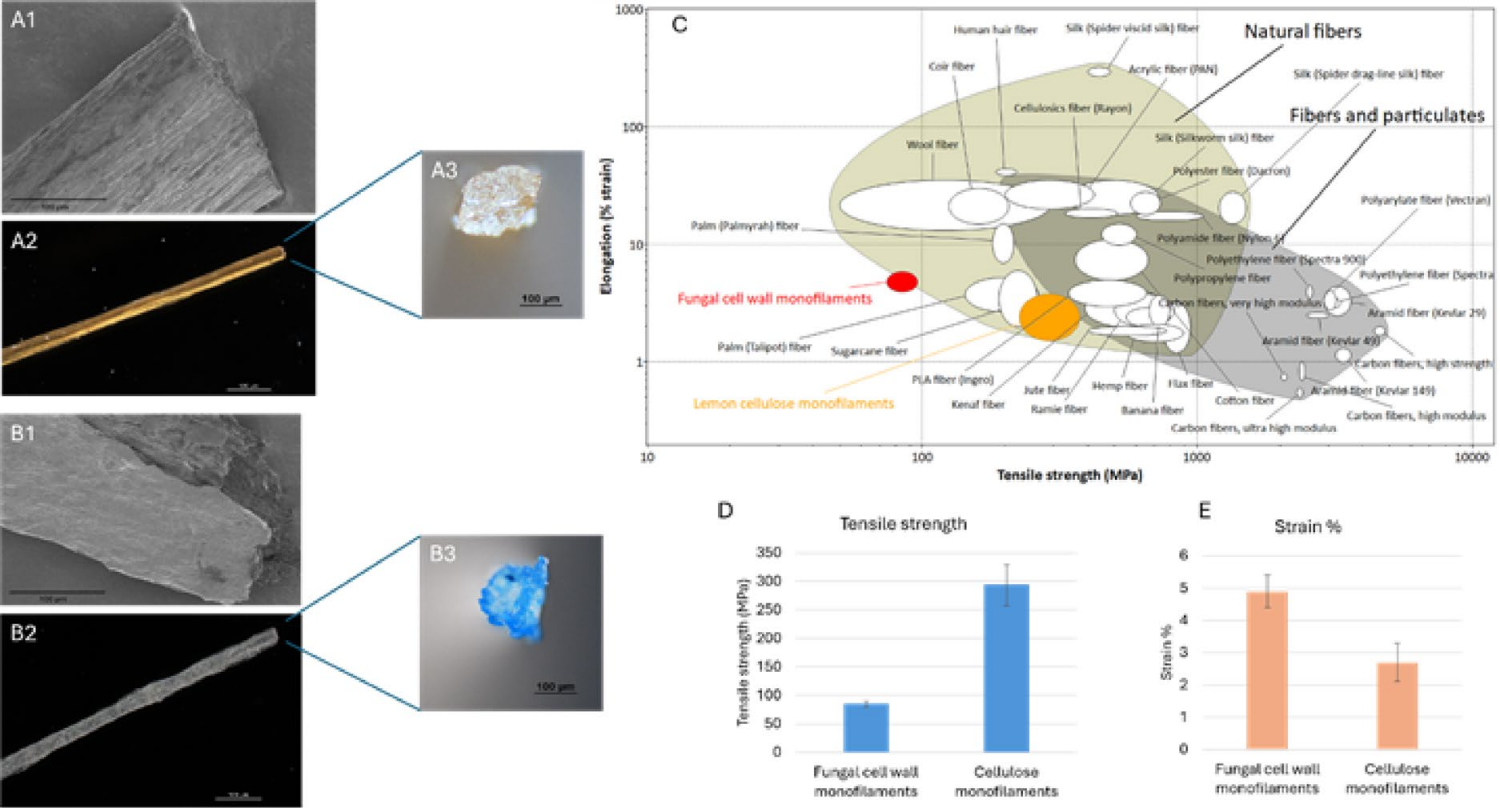
Optical and scanning electron microscopy images of **A** – Fungal monofilament and **B** – Cellulose monofilament (A1 and B1 – Surface morphology obtained from SEM (Scale bar 100 μm), A2 and B2 – Surface morphology obtained from Optical microscope (Scale bar 500 μm), A3 and B3 – Cross-section obtained from optical microscope (Scale bar 100 μm)). **C** – Ashby plot comparing the properties of the produced monofilaments with those of commercially available fibres (plotted with Granta EduPack 2021 R2 Version: 21.2.0). **D** & **E** – Tensile strength and strain % of the produced fungal and cellulose monofilaments.

In parallel, the preparation of the spinning dope for cellulose monofilament production followed a different approach. Here, sample no 14 (Fig. 4A) collected from cellulose grinding was concentrated by centrifugation. After two 30-minute centrifuge steps at 10,000 g, the pulp concentration reached 4 ± 0.5%. The concentrated pulp was used directly as a spinning dope without any modifications.

Figure 5: Optical and scanning electron microscopy images of A – Fungal monofilament and B – Cellulose monofilament (A1 and B1 – Surface morphology obtained from SEM (Scale bar 100 μm), A2 and B2 – Surface morphology obtained from Optical microscope (Scale bar 500 μm), A3 and B3 – Cross-section obtained from optical microscope (Scale bar 100 μm)). C – Ashby plot comparing the properties of the produced monofilaments with those of commercially available fibres (plotted with Granta EduPack 2021 R2 Version: 21.2.0). D & E – Tensile strength and strain % of the produced fungal and cellulose monofilaments.

According to our previous experiments on dry gel spinning, both spinning dope concentrations were adjusted to 4%, and the spun monofilaments were collected on a parafilm surface to obtain a stable monofilament^26^. Continuous monofilament spinning was possible with the produced fungal cell wall hydrogel and micro-nanocellulose spinning dopes. However, the spinning of the fungal cell wall hydrogel was notably smoother than that of cellulose. This could be due to the shear-thinning rheological profile described by Lindh, et al^26^. on dry gel spinning of fungal cell wall material of *Rhizopus delemar* grown on hydrolysed bread media. These characteristics facilitate continuous and more uniform filament formation with the fungal cell wall during the spinning process, resulting in improved processability compared to the comparatively more rigid and viscous cellulose suspensions.

The optical microscopy images (Fig. 5A2, A3, B2, and B3) and scanning electron microscopy images (Fig. 5A1 and B1) show the morphologies of the spun fungal and cellulose monofilaments, respectively. The fungal cell wall monofilament has a more organised structure, whereas the micro-nanocellulose monofilament is uneven with flattening of the contact side during drying. This morphological difference could be related to the smoothness of the dry gel spinning process described previously. The flattening of the monofilaments is common in dry gel spinning^26,50^ and those structural changes usually have a minimum effect on the mechanical performance.

The linear density of the produced monofilaments was calculated by manual weight measurements of 10 cm length monofilament pieces, followed by calculation. The obtained linear densities of fungal cell wall monofilaments and cellulose monofilaments are 270 dtex and 240 dtex, respectively. These values are in the same range as previously published fungal cell wall dry gel spun monofilament linear densities^26^. Conversion of the calculated linear density to yield indicated that 1 g of starting lemon peel waste could produce 0.69 m of fungal cell wall monofilaments and 4.23 m of cellulose monofilaments. Details of the yield calculations are available in the raw data file.

### Mechanical properties of the produced monofilaments

The tensile properties of the produced monofilaments were also evaluated. The highest tensile strength of 298.3 MPa (Fig. 5D) was achieved for the micro-nanocellulose monofilaments. This value is higher than the highest tensile strength values reported for previous nanocellulose dry gel-spun fibres by Hooshmand, et al. ^19,^Hooshmand, et al. ^51,^Shen, et al^52^., which are 260 MPa, 222.0 MPa, and 220 MPa, respectively (P values were more than 0.05 in all cases with the assumption of the number of replicates are not more than 3, hence considered as a non-significant increment). The strain % (elongation % at break) of the micro-nanocellulose monofilaments produced in this work is 2.65 (Fig. 5E), which is lower than the range of 4.5 to 8.3 reported by Hooshmand, et al^51^., but within the range of 2.6 to 4.9, as reported by Hooshmand, et al^19^.. In their research, Hooshmand, et al^51^. explained how the orientation and elongation were improved in the as-spun filaments by adding hydroxyethyl cellulose (HEC).

The fungal cell wall monofilaments exhibited a tensile strength of 85 MPa, which is more than four times less than that of the cellulose monofilaments. The higher tensile strength is an inherited property of cellulose-based fibres owing to the natural structural function of cellulose in plant cells^53,54^. On the other hand, chitosan, which is the main polymer component in fungal cell wall monofilaments, infamously lacks mechanical stability^55^. However, the tensile strength of the fungal cell wall monofilaments is higher than the 30–72.3 MPa range obtained previously with fungal cell wall material-based monofilaments produced using the wet-spinning method without post-treatment^13,14,31^. The strain % (elongation % at break) was within the range of 2.7 to 27.4 reported in the same research works. In the same study, higher tensile strength of 149 MPa and higher elongation % at break of 13.8% were achieved by treating fungal monofilaments with water and glycerol post-treatments, respectively, followed by 5% drawing. These post-treatments could be considered as a future work to improve the mechanical properties of the monofilaments produced in the present study as well.

Comparing the properties of the produced monofilaments with those of commercially available fibres is important to identify their feasibility for use in textile applications. Therefore, the produced monofilaments were placed in Ashby’s plots using Ansys Granta Edu Pack 2021 R2 Version: 21.2.0 software to compare their mechanical properties with those of commercially available textile fibres (Fig. 5C). Micro-nanocellulose-based monofilaments were placed on the natural fibre bubble and exhibited properties comparable to those of commercially used textile fibres. The tensile strength and strain % of the produced cellulose monofilaments were closely related to those of sugarcane, PLA, and palm fibres. The fungal cell wall monofilaments are placed on the border of the natural fibres and are related to the tensile strength of wool fibres. When considering the strain %, they were more related to palm and polylactic acid (PLA). Although these novel monofilaments show potential, their tensile strength and elongation characteristics can be further improved to match the performance of commonly used cellulose-based textile fibres, such as cotton and rayon. As of now, the fungal monofilaments could be used for medical textile applications such as wound dressings due to their antibacterial properties^31^. The cellulose monofilaments have potential for use in disposable protective clothing and nonwoven wipes, given their origin from a by-product and the biodegradability of chemically unmodified cellulose.

## Conclusion

This work demonstrates a holistic approach to lemon peel waste valorisation. The filamentous fungus *Rhizopus delemar* was cultivated successfully on the nutrient-rich liquids extracted from the lemon peel waste. The solid residues from the fungal cultivation step were used to purify the cellulose fractions. The quality of the purified cellulose fraction was analysed and confirmed using different analytical methods. The effectiveness of cellulose pulp fibrillation was confirmed using viscosity measurements together with SEM and AFM with evidence on nanofibrilation. Monofilaments were produced using both the fungal cell wall fraction and mechanically fibrillated, chemically unmodified cellulose through a cost-effective and environmentally friendly dry gel spinning process that eliminates the need for solvents and coagulants. The processes employed, including fungal cultivation, cellulose purification, and monofilament spinning, can be easily scaled up using pilot or industrial-scale equipment. The cellulose monofilaments exhibited properties comparable to those of commercial fibres, such as jute, PLA, and kenaf, while the fungal cell wall monofilaments exhibited acceptable properties in the natural fibre category. Overall, this study demonstrated the successful valorisation of low-value lemon peel waste into bio-based textile filaments through an environmentally friendly and resource-efficient process, offering a compelling model for sustainable material innovation within the circular economy framework. Nonetheless, limitations such as variability in feedstock composition, the absence of pilot scale processing and the need for comprehensive techno-economic and life cycle assessments were identified. Addressing these aspects in future research will be essential for translating this concept into scalable and commercially viable applications.

## Data Availability

The research data is saved in Figshare data repository and can be accessed via https://doi.org/10.6084/m9.figshare.30076024.v1.
